# Pädiatrische stationäre interdisziplinäre multimodale Schmerztherapie in Deutschland

**DOI:** 10.1007/s00482-023-00744-3

**Published:** 2023-08-29

**Authors:** Julia Wager, Boris Zernikow

**Affiliations:** 1 Deutsches Kinderschmerzzentrum, Vestische Kinder- und Jugendklinik Datteln, Datteln, Deutschland; 2 Lehrstuhl für Kinderschmerztherapie und Pädiatrische Palliativmedizin, Fakultät für Gesundheit, Department für Humanmedizin, Universität Witten/Herdecke, Witten, Deutschland

**Keywords:** Chronischer Schmerz, Multimodale Therapie/Wirksamkeit, Kinder, Jugendliche, Multiprofessionelle Versorgung, Chronic pain, Combined modality therapy/effectiveness, Children, Adolescents, Intensive interdisciplinary pain treatment

## Abstract

**Hintergrund:**

Schwer beeinträchtigende chronische Schmerzen betreffen etwa eine halbe Million Kinder in Deutschland. Bei fehlendem Ansprechen auf eine unimodale Therapie kann eine stationäre interdisziplinäre multimodale Schmerztherapie (IMST) in Erwägung gezogen werden.

**Ziel der Arbeit:**

In dieser Übersichtsarbeit wird die Versorgungslage der pädiatrischen stationären IMST in Deutschland beschrieben sowie aktuelle Evidenz zur Wirksamkeit dargestellt.

**Material und Methoden:**

Mithilfe einer systematischen Literatursuche wurden Studien identifiziert, die sich mit der Wirksamkeit der pädiatrischen stationären IMST in Deutschland befassen. Zudem wurden weiterführende Quellen genutzt, um Informationen über IMST-Angebote in Deutschland, deren Behandlungsprogramme, die Qualifikation der Behandelnden und Charakteristika der Patient:innen zu beschreiben.

**Ergebnisse:**

In Deutschland gibt es vier pädiatrische Schmerzzentren, die auf die stationäre IMST von Kindern und Jugendlichen spezialisiert sind. Die 3‑ bis 4‑wöchige multimodale Behandlung wird von einem multiprofessionellen Team angeboten und steht in der Regel Patient:innen bis zum 18. Lebensjahr zur Verfügung. Die Mehrzahl der Patient:innen ist weiblich. Die Wirksamkeit der pädiatrischen IMST in Deutschland wurde bis zu 4 Jahre nach der Behandlung untersucht. Positive Effekte zeigen sich sowohl für Schmerzeigenschaften als auch für die emotionale Belastung. Ergänzende Therapiemodule können die bestehenden Effekte noch weiter optimieren.

**Schlussfolgerung:**

Weitere Forschung zur Wirksamkeit der IMST in Deutschland ist wichtig, um das Behandlungsangebot weiterentwickeln und optimieren zu können.

## Abstract (en)

**Background:**

Severely disabling chronic pain affects approximately half a million children in Germany. If there is a lack of response to unimodal treatment, an inpatient interdisciplinary multimodal pain treatment (IMPT) can be considered.

**Objective:**

This review article describes the supply situation of pediatric inpatient IMPT in Germany and presents the current evidence on the effectiveness.

**Material and methods:**

Based on a systematic literature search, studies addressing the effectiveness of pediatric inpatient IMPT in Germany were identified. In addition, further sources were used to extract information on pediatric IMPT treatment centers in Germany in order to describe the treatment approaches, the qualification of personnel and characteristics of patients.

**Results:**

There are four pediatric pain centers in Germany that are specialized in inpatient IMPT for children and adolescents. Treatment duration ranges between 3 and 4 weeks. The multimodal treatment is carried out by a multiprofessional team and is generally available for patients up to 18 years. The majority of patients are female. The effectiveness of pediatric IMPT in Germany was investigated up to 4 years after treatment. Positive effects were shown for pain characteristics and also for the emotional burden. Additional treatment modules can facilitate further optimization of treatment effects.

**Conclusion:**

Further research on the effectiveness of IMPT in Germany is important in order to refine and optimize the available treatment programs.

In Deutschland erleben nach aktuellen epidemiologischen Daten etwa 30 % der Kinder und Jugendlichen Schmerzen, die mindestens 3 Monate anhalten und mindestens wöchentlich auftreten [33, 37]. Bei etwa 8 % gehen diese chronischen Schmerzen mit weiteren schweren Symptomen einher, beispielsweise mit einer emotionalen oder starken funktionellen Beeinträchtigung, die sowohl den Alltag als auch den Schulbesuch erschwert [27]. Auf die deutsche Gesamtbevölkerung hochgerechnet sind fast eine halbe Million Kinder und Jugendliche zwischen 10 und 17 Jahren von diesen schwer beeinträchtigenden chronischen Schmerzen betroffen [5].

Da schwer beeinträchtigende chronische Schmerzen das Entwicklungspotenzial der Betroffenen reduzieren [29], die Wahrscheinlichkeit für ein Anhalten der Schmerzproblematik bis ins Erwachsenenalter hoch ist und chronischer Schmerz bei Kindern zudem die Entwicklung weiterer psychischer und psychosomatischer Beschwerden im Erwachsenenalter begünstigt [2], ist eine frühzeitige sowie wirksame Behandlung unbedingt notwendig.

Bei Kindern mit chronischem Schmerz ist eine frühzeitige und wirksame Behandlung unbedingt notwendig

Betroffene nehmen in der Regel bei einer wiederkehrenden Schmerzproblematik zunächst Kontakt mit der Kinder- und Jugendärzt:in auf. In der pädiatrischen Primärversorgung können die meisten Betroffenen ausreichend gut versorgt werden; ihre Schmerzproblematik remittiert innerhalb eines halben Jahres [39]. Einige Patient:innen erleben jedoch eine weitere Eskalation der Beschwerden. Sie sollten möglichst frühzeitig an eine spezialisierte Versorgungseinrichtung überwiesen werden. Bereits nach 3 Monaten ist erkennbar, ob eine Patient:in ausreichend gut auf die Behandlung in der Primärversorgung anspricht [39]. Tritt in diesem Zeitfenster keine deutliche Besserung ein, sollte eine Therapieintensivierung, beispielsweise in Form einer stationären interdisziplinären multimodalen Schmerztherapie (IMST), in Erwägung gezogen werden. In Deutschland sind die Zugangsvoraussetzungen sowie die Strukturkriterien der IMST klar durch den Operationen- und Prozedurenschlüssel im Gesundheitswesen OPS 8‑918 definiert [3].

In der vorliegenden Übersichtsarbeit wird basierend auf einer systematischen Literaturrecherche die Versorgungslage der pädiatrischen stationären IMST in Deutschland beschrieben sowie aktuelle Evidenz zur Wirksamkeit dargestellt.

## Material und Methoden

### Literatursuche und Einschlusskriterien

In diese Übersichtsarbeit gehen Studien ein, die sich mit der Wirksamkeit der pädiatrischen stationären IMST in Deutschland befassen. Basis für die eingeschlossenen Studien ist eine aktuelle systematische Übersichtsarbeit und Metaanalyse von Claus et al. [4], die internationale pädiatrische IMST-Programme berücksichtigt. Aus den insgesamt 51 eingeschlossenen Studien (siehe „Study IDs“ auf https://osf.io/jnzq5/) wurden diejenigen Publikationen ausgewählt, die sich mit IMST-Programmen in Deutschland befassen; dies waren insgesamt 16 Publikationen [7, 9, 13, 15, 17–23, 28, 31, 34, 36, 41]. Zudem wurde die Literatursuche von Claus et al. [4] am 28. Februar 2023 in PubMed (MEDLINE) aktualisiert (siehe „Search Strategy“ auf https://osf.io/jnzq5/). Bei dieser erneuten Suche wurden 300 Publikationen identifiziert, deren Abstracts nach den Kriterien aus der Studie von Claus et al. [4] gescreent wurden:

- Die Intervention wurde von mindestens 3 Gesundheitsberufen durchgeführt.
- Die Behandlung erfolgte stationär oder tagesstationär.
- Die Studien sind auf Englisch oder Deutsch publiziert.
- Die Patient:innen sind ≤ 22 Jahre alt.
- Die Patient:innen leiden an schweren und beeinträchtigenden primären Schmerzen.

Als zusätzliches Einschlusskriterium wurde auch hier als Lokalisation des IMST-Programms „Deutschland“ ergänzt. Für 10 Publikationen wurde der Volltext gescreent und schließlich wurden 3 zusätzliche Studien eingeschlossen [11, 12, 38].

Somit werden insgesamt 19 Publikationen berücksichtigt. Für die Darstellung der Wirksamkeit werden nur Studien herangezogen, deren Patient:innenpopulationen sich nicht überschneiden [9, 12, 15, 20, 28, 34, 41]. Neben den identifizierten Publikationen werden weiterführende Quellen wie publizierte Therapiemanuale, Lehrbücher, weiterführende Fachartikel und Internetseiten für eine umfassende Darstellung der pädiatrischen IMST in Deutschland genutzt. Diese Quellen wurden mittels unsystematischer Handsuche detektiert.

### Datenextraktion

Aus den Studien wurden Informationen zu folgenden Inhalten extrahiert:

- Name, Ort und Eigenschaften der Schmerzeinrichtung
- Charakteristika des Behandlungsprogramms/Behandlungsmodule
- Qualifikation der Behandelnden
- Charakteristika der Patient:innen
- Wirksamkeit der IMST-Behandlung

Diese Informationen werden im Folgenden übersichtlich dargestellt.

## Ergebnisse

### Pädiatrische IMST-Programme in Deutschland

Im Jahr 2003 wurde eine stationäre IMST für Kinder und Jugendliche am Zentrum für Schmerztherapie junger Menschen in Garmisch-Partenkirchen etabliert [23]. Aktuell werden nach eigenen Angaben mehr als 500 Patient:innen jährlich behandelt; der Schwerpunkt der Behandlung liegt auf muskuloskeletalen Schmerzen [23], wobei das Behandlungsspektrum auch chronische Kopf- und Bauchschmerzen umfasst [40].

Das erste in der für diese Übersichtsarbeit identifizierten Literatur erwähnte pädiatrische IMST-Programm in Deutschland ist am Deutschen Kinderschmerzzentrum in Datteln an der Vestischen Kinder- und Jugendklinik – Universität Witten/Herdecke lokalisiert [7]. Die stationäre IMST wurde hier im Jahr 2005 erstmalig auf einer eigens dafür eingerichteten Station angeboten [6]. Aktuell werden nach Angaben auf der Website mehr als 300 Kinder und Jugendliche pro Jahr stationär behandelt. Die IMST wird für Kinder ab 7 Jahren beschrieben [7]. Regulär können in der pädiatrischen Einrichtung Patient:innen bis zu einem Alter von 17 Jahren behandelt werden. Es gibt jedoch auch Behandlungsangebote bis zum 25. Lebensjahr [32].

In der Studienregistrierung (DRKS-ID: DRKS00015230) einer im Jahr 2021 veröffentlichten Studie werden zudem Augsburg und Stuttgart als Behandlungszentren für die pädiatrische IMST erwähnt [11]. Sowohl das Bayerische Kinderschmerzzentrum am Universitätsklinikum Augsburg [1] als auch das Kinderschmerzzentrum Baden-Württemberg am Klinikum Stuttgart [26] bieten eine stationäre IMST für Kinder und Jugendliche an. Am Kinderschmerzzentrum Baden-Württemberg erhalten nach Angaben auf der Website jährlich etwa 260 Patient:innen (Stand 2016) im Alter von 6 bis 18 Jahren eine stationäre IMST.

### Charakteristika der IMST bzw. der Behandlungsmodule

Die Behandlungsprogramme in Augsburg, Datteln und Stuttgart werden manualisiert durchgeführt [10, 11]. Erstmalig wurde dieser Therapieansatz im Jahr 2006 publiziert [7]. Die stationäre IMST ist eine hochfrequente schmerztherapeutische Intensivtherapie mit einer Dauer von 3 bis 4 Wochen und einem täglichen Behandlungsumfang von etwa 8 h [8]. Pro Woche haben die Patient:innen unter anderem 3 bis 4 psychotherapeutische Einzelsitzungen, ein Familiengespräch sowie 2 Gruppentherapien und besuchen täglich die Klinikschule. Während eines stationären Aufenthalts finden zudem 2 Belastungserprobungen statt, bei denen die Patient:in für das Wochenende beurlaubt wird und in der Regel am darauf folgenden Montag die Heimatschule besucht. Auch die Erziehungs- und Sorgeberechtigten haben neben den Familiengesprächen die Möglichkeit, in die Behandlung eingebunden zu werden, indem sie auf der Station hospitieren, um zu sehen, wie das Pflege- und Erziehungsteam mit ihrem schmerzkranken Kind umgeht. So sollen sie beispielsweise lernen, wie aktive Schmerzbewältigungsmaßnahmen ganz konkret umgesetzt werden.

Insgesamt werden im manualisierten Behandlungsprogramm 6 Module beschrieben [8]:

- Modul 1: Edukation und Zielklärung
- Modul 2: Trainieren von Schmerzbewältigungsstrategien
- Modul 3: Therapie komorbider emotionaler Symptomatiken
- Modul 4: Familientherapie
- Modul 5: Optionale Interventionen, beispielsweise medikamentöse Therapie, Physiotherapie, Milieutherapie, Musik- und Kunsttherapie, Sozialberatung
- Modul 6: Rückfallprophylaxe, Therapieabschluss inklusive der Planung der Nachbetreuung

Diese Module werden für jede Patient:in individuell ausgestaltet. So erhalten beispielsweise Patient:innen mit muskuloskeletalen Schmerzen mehr physiotherapeutische Behandlung als beispielsweise Patient:innen mit Kopf- oder Bauchschmerzen. Die Therapie einer komorbiden emotionalen Symptomatik wird individuell an das jeweilige Störungsbild angepasst und variiert in ihrem Umfang ebenfalls je nach Schwere der psychischen Beeinträchtigung [8].

Nach abgeschlossener stationärer Behandlung erhalten die Patient:innen das Angebot einer poststationären Betreuung [11]. Diese Nachbetreuung umfasst eine Wiedervorstellung in der Schmerzambulanz 3 und 6 Monate nach der stationären Entlassung. Im Rahmen einer Studie wurde das reguläre Nachsorgeregime temporär erweitert. Familien wurden für einen Zeitraum von 6 Monaten von einem multiprofessionellen Team unter Leitung einer Sozialarbeiter:in begleitet [11]. Die Begleitung der Patient:innen und ihrer Familien erfolgte nach dem Konzept der sozialmedizinischen Nachsorge und zielte darauf ab, nach der IMST das neu Erlernte in den Alltag zu übertragen. Es wird so viel Unterstützung wie nötig, jedoch so wenig wie möglich angeboten. Unterstützung erhalten die Patient:innen bei der Initiierung ambulanter Maßnahmen, wie Physiotherapie, ambulanter Psychotherapie oder Kontrollterminen bei Fachärzt:innen, bei der Etablierung einer aktiven Alltagsstruktur mit regelmäßigem Schulbesuch sowie beim Anwenden empfohlener Therapien wie Ablenkungs- und Entspannungstechniken oder bei Medikamenteneinnahmen.

Für die stationäre IMST in Garmisch-Partenkirchen werden ähnliche Behandlungsmodule wie im manualisierten Konzept beschrieben [23]. Nach Angaben aus dem Referenzbericht 2021 werden die Patient:innen jedoch nicht nach OPS 8‑918 abgerechnet [16]. Die Behandlung wird von einem multiprofessionellen Team angeboten und umfasst Edukation, ärztliche Anamnese und Diagnostik, medikamentöse Behandlung, psychologische Gruppen- und Einzeltherapien, Behandlungen der physikalischen Abteilung, wie ergonomisches Training, Atemtherapie oder Osteopathie, Klinikschule sowie eine Mitbehandlung durch den Pflege- und Sozialdienst. Die Dauer der stationären IMST beträgt auch in Garmisch-Partenkirchen etwa 3 Wochen [23].

### Qualifikation der Behandelnden

Das Kernteam der in Augsburg, Datteln und Stuttgart durchgeführten manualisierten Therapie [8] umfasst

- das Pflege- und Erziehungsteam,
- Kinder- und Jugendmediziner:innen bzw. Kinder- und Jugendpsychiater:innen,
- weitere medizinische Disziplinen (beispielsweise Kinderradiolog:innen zum Ausschluss sekundärer Schmerzerkrankungen oder zur Edukation anhand radiologischer Befunde) sowie
- Kinder- und Jugendlichenpsychotherapeut:innen.

Weitere pädagogische bzw. therapeutische Disziplinen unterstützen die stationäre Arbeit beispielsweise durch

- Physiotherapie,
- Musiktherapie,
- Kunsttherapie,
- Klinikschule,
- Motopädie und
- Sozialarbeit.

In Garmisch-Partenkirchen bilden folgende Disziplinen das Behandelndenteam:

- Ärzt:innen,
- Physiotherapeut:innen,
- Ergotherapeut:innen,
- Psycholog:innen/Psychotherapeut:innen,
- Sozialpädagog:innen,
- spezialisierte Pflegekräfte sowie
- Mitarbeitende der Klinikschule und des Sozialdiensts [23].

Der interdisziplinäre Austausch stellt einen wesentlichen Bestandteil der Zusammenarbeit dar, damit in der Behandlung einzelne Aspekte zusammengeführt werden und so die Patient:innen ganzheitlich betrachtet und therapiert werden [23].

### Charakteristika der Patient:innen

Zugangsvoraussetzungen für eine stationäre pädiatrische IMST ergeben sich aufgrund des OPS 8‑918 [3], der altersunabhängig Anwendung findet. Die Patient:innen erhielten die Hauptdiagnose F45.4 bzw. F45.41 nach Internationaler statistischer Klassifikation der Krankheiten und verwandter Gesundheitsprobleme (ICD-10; [11]). Zudem haben die Patient:innen eine reduzierte Lebensqualität, beispielsweise erfasst mit dem KIDSCREEN-27 ([30]; M = 96), gehen nicht regelmäßig zur Schule (durchschnittlich 5–6 Schulfehltage in den letzten 4 Wochen), vorherige unimodale Schmerztherapien sind fehlgeschlagen, oft besteht ein Medikamentenfehlgebrauch und viele Patient:innen leiden an einer schmerzunterhaltenden psychischen Begleiterkrankung, wie einer Depression oder Angststörung, bzw. an einer somatischen Begleiterkrankung, beispielsweise einer juvenilen idiopathischen Arthritis [11]. In Garmisch-Partenkirchen haben 36 % der Patient:innen ein sekundäres Schmerzsyndrom [23]. Genaue Häufigkeitsangaben zu fehlgeschlagenen Therapien, Medikamentenfehlgebrauch und psychischen Begleiterkrankungen sind den vorliegenden Quellen nicht zu entnehmen. Hinweise zur emotionalen Beeinträchtigung der Patient:innen geben jedoch Screeningfragebögen, wie beispielsweise die Revised Child Anxiety and Depression Scale (RCADS; [35]; Depressionsscore: M = 11, Angstscore: M = 28; [11]).

Patient:innen, die in Deutschland eine pädiatrische IMST erhalten, sind im Schnitt 14 Jahre alt und vornehmlich weiblich (zwischen 72 und 80 %; [11, 23]). Mehr als 95 % der Patient:innen haben keinen Migrationshintergrund [11]. Während in Garmisch-Partenkirchen vor allem muskuloskeletale Schmerzen behandelt werden, ist der Behandlungsschwerpunkt in den Schmerzzentren in Augsburg, Datteln und Stuttgart etwas anders gewichtet. Hier haben etwa 70 % der Patient:innen Kopfschmerzen, gefolgt von muskuloskeletalen Schmerzen (etwa 45 %) und Bauchschmerzen (etwa 25 %), wobei bei mehr als einem Drittel mehrere Schmerzorte vorliegen [11]. Nur etwa 30 % haben ihre Schmerzen seit weniger als einem Jahr; 35 % hingegen sogar länger als 3 Jahre [11].

### Wirksamkeit der IMST-Behandlung

Die Wirksamkeit der pädiatrischen IMST wurde bislang in einer randomisierten, kontrollierten Studie in Deutschland überprüft. Es zeigte sich eine deutliche Wirksamkeit zum Zeitpunkt der IMST-Entlassung im Vergleich mit einer Wartekontrollgruppe [20]. Von den Patient:innen, die zum Vergleichszeitpunkt bereits die Therapie erhalten hatten, erlebten 55 % eine allgemeine Verbesserung, das heißt, sie hatten sich in keiner schmerzbezogenen Variable (Schmerzintensität, Beeinträchtigung im Alltag, Schulfehltage) verschlechtert und erlebten eine klinisch signifikante Verbesserung mindestens in schmerzbezogener Beeinträchtigung oder Schulfehltagen. Lediglich 14 % in der Wartekontrollgruppe erzielten diese allgemeine Verbesserung. Darüber hinaus zeigte sich zum Zeitpunkt der stationären Entlassung eine signifikante Verbesserung der Depressivität und des schmerzbezogenen Katastrophisierens, während die Wartekontrollgruppe sich nicht signifikant verbesserte [20].

Weitere Studien beschreiben das Therapieoutcome bis zu 4 Jahre nach Entlassung aus der IMST, können die Ergebnisse jedoch nicht mit einer Kontrollgruppe vergleichen. Diese Studien belegen bis zu 12 Monate nach der stationären IMST eine moderate bis starke Reduktion der Schmerzintensität, der Beeinträchtigung im Alltag sowie der Schulfehltage [9, 20]. Die positiven Effekte konnten auch 4 Jahre nach der stationären IMST aufrechterhalten werden [41]. Die Betrachtung eines mehrdimensionalen Endpunkts, des Chronic Pain Grading (CPG) zur Einschätzung des Schmerzschweregrads, zeigt ebenfalls eine geringe, jedoch signifikante Besserung der Schmerzsymptomatik 12 Monate nach Erstvorstellung [34].

Auch die emotionale Beeinträchtigung bessert sich nach der stationären IMST. Depressions- und Angstsymptome nehmen 3 bzw. 6 Monate nach der stationären IMST ab [9, 20]. Diese positiven Effekte sind weitestgehend auch 12 Monate [9, 20] und sogar bis zu 4 Jahre später [41] noch abbildbar. Auch der passive Umgang mit Schmerzen reduziert sich 3 Monate nach der IMST moderat und die Suche nach sozialer Unterstützung bei Schmerzen nimmt geringfügig ab; die Effekte bleiben bis zu 12 Monate stabil [9]. Das schmerzbezogene Katastrophisieren ist ebenfalls bis zu 4 Jahre nach einer stationären IMST reduziert [20, 41]. Einschränkend ist darauf hinzuweisen, dass alle bisherigen Studien zur Wirksamkeit der pädiatrischen IMST in Deutschland am Standort Datteln durchgeführt wurden.

Ergänzend zur patientenbezogenen Wirksamkeit wurden auch ökonomische Outcomes im Kontext der pädiatrischen IMST betrachtet. Nach Angaben der Eltern am Standort Datteln nahmen Patient:innen über einen Zeitraum von bis zu 4 Jahren nach IMST-Entlassung signifikant weniger gesundheitliche Leistungen in Anspruch als vor der Therapie, beispielsweise in Bezug auf hausärztliche Versorgung, Psychotherapie, Physiotherapie und Osteopathie [20, 41]. Zudem reduzierten sich die Arbeitsfehltage der Eltern (vor der Therapie im Median 4 Tage innerhalb von 6 Monaten; nach der Therapie im Median 0 Arbeitsfehltage; [20]). Diese positiven ökonomischen Effekte zeigten sich vor allem für diejenigen Patient:innen, die eine allgemeine Verbesserung der Schmerzsituation erreichten [20, 41].

Die Kosten für Krankenkassen reduzieren sich im ersten und erneut im zweiten Jahr nach Therapie

Krankenkassendaten, die ein Jahr vor und bis zu 2 Jahre nach der Therapie analysiert wurden, bestätigen dieses Muster [28]. Die Gesamtkosten für Krankenkassen reduzieren sich im ersten und dann erneut im zweiten Jahr nach der Therapie signifikant. Dies liegt vor allem an einer Reduktion der allgemeinen stationären Behandlungskosten nach der IMST, die sich auf 50 % der Gesamtkosten belaufen. Kosten im ambulanten Sektor, die etwa 30 % der Gesamtkosten darstellen, reduzieren sich erst im zweiten Jahr nach der IMST. Dies liegt unter anderem daran, dass im Jahr nach der IMST die Inanspruchnahme einer ambulanten Psychotherapie zunächst stabil bleibt. Kosten für Heil- und Hilfsmittel reduzieren sich bereits im Jahr nach der IMST; Kosten für Medikamente verändern sich nicht signifikant [28].

Weitere randomisierte, kontrollierte Studien befassen sich mit der Ergänzung der IMST durch neue Therapiemodule. In einer dieser Studien wurde die Therapieerweiterung um ein interozeptives Expositionsverfahren, genannt Schmerzprovokation, mit einer Ergänzung um ein Entspannungsverfahren, die progressive Muskelrelaxation, verglichen. In dieser Studie, die ebenfalls in Datteln durchgeführt wurde, zeigte sich keine allgemeine Überlegenheit des Expositionsverfahrens gegenüber der Entspannung [15]. Explorative Analysen zeigten jedoch, dass für Patient:innen mit einer hohen Ausprägung schmerzbezogener Ängste sowie für Patient:innen mit Bauchschmerzen das Expositionsverfahren in Bezug auf schmerzbezogene Ängste effektiver ist als das Entspannungsverfahren.

Eine weitere randomisierte, kontrollierte Studie, die multizentrisch in den Kinderschmerzzentren Augsburg, Datteln und Stuttgart durchgeführt wurde, zeigte den zusätzlichen Nutzen einer intensiveren Nachbetreuung der Patient:innen und Familien. Durch ein 6‑monatiges Programm, das bereits im Abschnitt „Charakteristika der IMST bzw. der Behandlungsmodule“ beschrieben wurde, konnte eine deutliche Verbesserung im Therapieansprechen erzielt werden, gemessen anhand des Schmerzschweregrads, der Lebensqualität, Selbstwirksamkeit sowie Depressivität und Ängstlichkeit [12]. Ein Jahr nach Entlassung litten in der Gruppe, die die zusätzliche Nachsorge erhielt, nur noch 40 % an chronischen Schmerzen, in der Gruppe mit Standardnachversorgung 70 % [12].

## Diskussion

In dieser Übersichtsarbeit werden die Durchführung und die bestehende Evidenz zur Wirksamkeit der stationären IMST für Kinder und Jugendliche in Deutschland beschrieben. Es fließen Daten von vier Kinderschmerzzentren in Deutschland ein. In einer aktuellen Publikation zur ambulanten und stationären Versorgungssituation von Kindern und Jugendlichen mit chronischen Schmerzen in Deutschland wird berichtet, dass insgesamt elf Einrichtungen eine stationäre Schmerztherapie für diese Altersgruppe anbieten [24]. Diese Angaben basieren auf einer Selbstauskunft der behandelnden Einrichtungen. Welche dieser Einrichtungen eine an OPS-Standards orientierte IMST anbieten, ist aus den Daten nicht ersichtlich. Da bei der systematischen Suche lediglich die vier in den Ergebnissen beschriebenen Einrichtungen identifiziert wurden, ist jedoch davon auszugehen, dass diese Zentren federführend in Behandlung und Forschung der IMST für Kinder und Jugendliche sind.

Angesichts der Häufigkeit chronischer Schmerzen sollten mehr Behandlungszentren eingerichtet werden

Die hohe Zahl der Kinder und Jugendlichen mit einem schwerwiegenden chronischen Schmerzproblem [27] und die geringe Anzahl zur Verfügung stehender Therapieplätze in den in dieser Arbeit identifizierten spezialisierten Zentren weist auf eine deutliche Diskrepanz zwischen Bedarf und Angebot hin. Es wäre wünschenswert, dass weitere spezialisierte Behandlungszentren für die Betroffenen eingerichtet werden. Weiterhin bilden weniger spezialisierte Strukturen, die in diesem Beitrag nicht adressiert wurden, eine wichtige Ergänzung der Versorgungsstruktur in Deutschland. Hier sind im Speziellen ambulante Angebote zu nennen ebenso wie stationäre Angebote, die im Rahmen der pädiatrischen Krankenhausversorgung eine psychosomatische oder eine kinder- und jugendpsychiatrische Komplexbehandlung anbieten. Auch ambulante psychotherapeutische Angebote von speziell ausgebildeten Kinder- und Jugendlichenpsychotherapeut:innen stellen einen wichtigen Teil der Versorgungsstruktur dar.

Die Behandlungsprogramme für pädiatrische IMST in Deutschland sind mit denen internationaler Behandlungszentren vergleichbar. Zwar gibt es Unterschiede in der konkreten Ausgestaltung von Therapiebestandteilen, jedoch bieten alle Einrichtungen psychologische und psychotherapeutische Module, medizinische Interventionen wie Medikation oder Untersuchungen, Physiotherapie und soziale Therapiemodule wie Familien- oder Elterngespräche sowie Gruppentherapien an [4]. Besonders hervorzuheben ist, dass drei der vier deutschen Behandlungszentren ihre IMST am selben Behandlungsmanual orientieren [10]. Dies ermöglicht die Durchführung multizentrischer Studien beispielsweise zur Untersuchung der Wirksamkeit ergänzender Therapiemodule [11].

Die Charakteristika der pädiatrischen Patient:innen, die eine stationäre IMST erhalten, verdeutlichen die stärkere Betroffenheit von Mädchen im Vergleich zu Jungen. Dies entspricht Erkenntnissen aus epidemiologischen Studien [25, 27], sodass diese Geschlechterverteilung wohl keinen Hinweis auf mögliche Zugangsbarrieren darstellt. Ebenso zeigen epidemiologische Studien eine Zunahme des Schmerzschweregrads mit dem Alter [25, 27], weshalb das durchschnittliche Alter der Patient:innen einer stationären IMST bei etwa 14 Jahren liegt. Auffällig ist der sehr geringe Anteil von Patient:innen mit Migrationshintergrund. Da bislang keine Hinweise vorliegen, dass das Risiko einer schweren Schmerzstörung bei Kindern und Jugendlichen mit Migrationshintergrund geringer ist [27], deutet die vergleichsweise geringe Anzahl in den beschriebenen Patient:innenpopulationen entweder auf eine selektive Studienteilnahme oder Zugangsbarrieren in der Inanspruchnahme hin. Spezifische Angebote, die neben sprachlichen Hürden auch kulturspezifische Besonderheiten adressieren, könnten für diese Zielgruppe das Angebot optimieren.

Auffällig ist der sehr geringe Anteil von Patient:innen mit Migrationshintergrund

Die Wirksamkeit in den deutschen Studien ist im internationalen Kontext vergleichbar. Betrachtet man die Heterogenität in der Metaanalyse von Claus et al. [4], wird deutlich, dass die deutschen Studien nur bezüglich der Schmerzintensität und der Alltagsbeeinträchtigung abweichen. In einer Studie zeigt sich eine stärkere Reduktion der Schmerzintensität im internationalen Vergleich [9] und in allen deutschen Studien, die in die Metaanalyse eingeschlossen wurden, ist die Reduktion der Beeinträchtigung geringer, was jedoch auch auf ein Artefakt des Erhebungsinstruments zurückzuführen sein kann [4]. Insgesamt weisen die in diesem Beitrag beschriebenen Wirksamkeitsstudien jedoch auf eine sehr gute Wirksamkeit bis zu 4 Jahre nach der IMST hin. Eine weitere Studie, die in der vorliegenden Arbeit aufgrund fehlender Baseline-Daten nicht in die Wirksamkeitsanalyse aufgenommen wurde, vergleicht den Gesundheitszustand ehemaliger Patient:innen 7 Jahre nach IMST mit dem einer bezüglich Alter und Geschlecht vergleichbaren Population [38]. Hier zeigt sich, dass viele der ehemaligen pädiatrischen Schmerzpatient:innen, die eine IMST erhalten haben, 7 Jahre nach der Behandlung kein relevantes Schmerzproblem mehr haben. Fast 60 % sind vollständig schmerzfrei; weitere 27 % erleben nur eine geringe schmerzbezogene Beeinträchtigung. Trotz dieser positiven Entwicklung berichten die ehemaligen Patient:innen unabhängig vom Vorhandensein chronischer Schmerzen eine schlechtere mentale und physische Gesundheit als die Vergleichsgruppe [38]. Die Ergebnisse zur intensiveren Nachsorge nach einer pädiatrischen IMST [11, 12] könnten wegweisend sein, um auch langfristig noch bessere Therapieoutcomes zu erzielen.

Trotz vielversprechender Ergebnisse der bisher durchgeführten Studien sind für eine abschließende Bewertung der Wirksamkeit der pädiatrischen stationären IMST weitere methodisch hochwertige Studien erforderlich. Besonders relevant sind in diesem Kontext randomisierte, kontrollierte Studien, die die Wirksamkeit der IMST bzw. einzelner Module belegen. Eine große Herausforderung ist es, Studien so zu konzipieren, dass die methodische Güte eine hohe Zuverlässigkeit der Evidenz erlaubt [14].

## Fazit für die Praxis

- Die stationäre interdisziplinäre multimodale Schmerztherapie (IMST) wird in Deutschland nur an einigen wenigen spezialisierten Zentren angeboten.
- Die Wirksamkeit wurde bereits in vielen Studien adressiert und entspricht der Wirksamkeit in internationalen Studien.
- Weitere Studien, die mithilfe eines randomisierten, kontrollierten Designs neue Erkenntnisse zur spezifischen Wirksamkeit der IMST für Kinder und Jugendliche generieren, sind wünschenswert, um eine breitere und zuverlässigere Evidenzbasis zu schaffen sowie ergänzende Therapiemodule weiterzuentwickeln.
